# Supplementary material for: In situ observation of picosecond polaron self-localisation in α-Fe2O3 photoelectrochemical cells
Source: Nat Commun. 2019 Sep 3;10:3962. doi: 10.1038/s41467-019-11767-9 (PMC6722133; doi:10.1038/s41467-019-11767-9)
Supplement: Supplementary file 1 — Supporting Information [file 41467_2019_11767_MOESM1_ESM.pdf]

Supplementary Information for

In situ observation of picosecond polaron self-localisation in α-Fe_2O_3 Photoelectrochemical Cells

Pastor *et al.*

Supplementary Figures

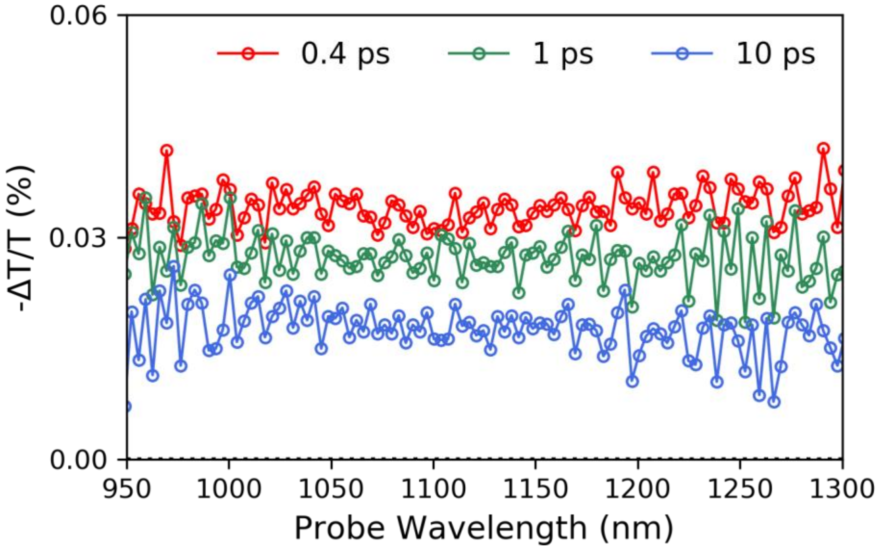

Supplementary Figure 1. **Transient Absorption spectrum of α-Fe_2O_3.** TA of α-Fe_2O_3 in the NIR region at different times after photoexcitation. In this region the spectrum is structureless before and after the formation of polaronic state complicating the interpretation of the TA data in the absence of complementary methods.

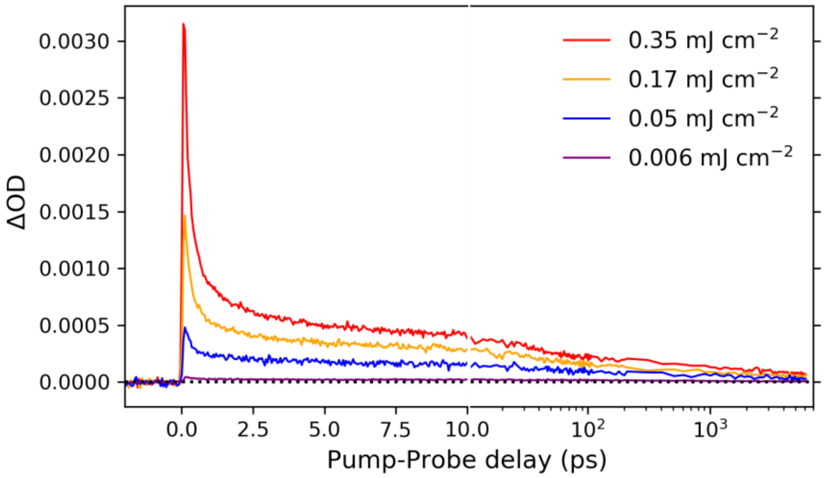

Supplementary Figure 2. **Fluence dependence of the Transient Absorption kinetics**. TA decay kinetics of α-Fe_2O_3 at different pump (400 nm) intensities, probed at 1200 nm.

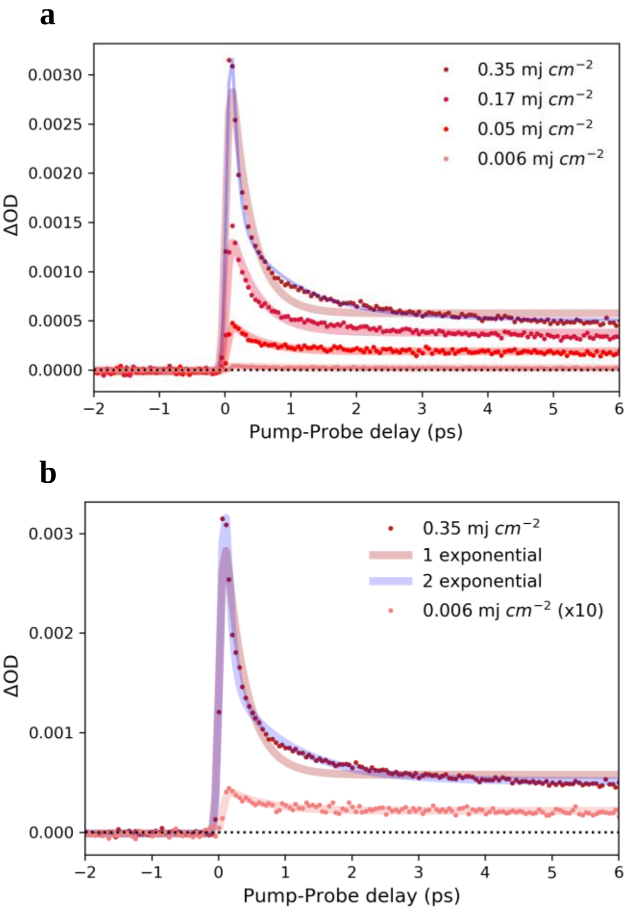

Supplementary Figure 3. **Fluence dependence of the TA at early times.** (**a**) TA decay kinetics of α-Fe_2O_3 at different pump (400 nm) intensities probed at 1200 nm with fits shown as solid lines. The decay time constant are: 0.308±0.014 (0.35 $mJcm^{-2}$); 0.406±0.019 (0.17 $mJcm^{-2}$); 0.43±0.04 (0.05 $mJcm^{-2}$); 0.54±0.07 (0.006 $mJcm^{-2}$) (**b**) Comparison of the highest and lowest intensities. At higher power we find that the data fits best a two exponential model with time constants: 0.077±0.006 ps and: 0.92±0.05 ps. This points towards non-germinate recombination becoming relevant at higher-intensities, although further work is required to identify this process and its relevance for photoelectrochemical cell activity. To obtain an estimate of the decay time constant (τ) in the TA data we fitted the data with a convolution of Gaussian and exponential decays of the form:

$$\mathrm{F(x)} = \int_{-\infty}^{\infty} \frac{e^{-\frac{t^2}{2\sigma^2}}}{\sigma\sqrt{2\pi}} \left[B + \left\{ \sum_{i=1}^{n} H_i e^{-((x-x_o)-t)/\tau_i} \right\} \right] \partial t + C$$

Where σ is the variance, n is the number of decay exponentials (one or two in our case), B is a step function and H_i and C are constants. Both B and the exponential response are evaluated to zero if x-x_o-t <0.

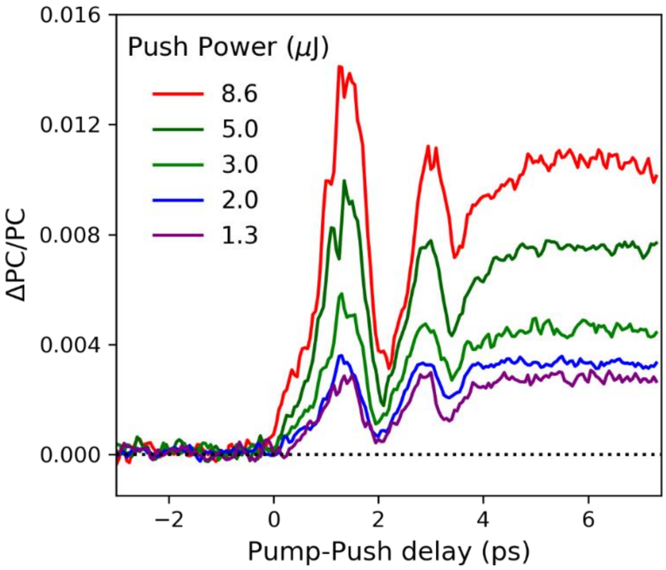

Supplementary Figure 4. **Push intensity dependence of the PPPC kinetics.** Pump-push-photocurrent (PPPC) response monitoring the change in the photocurrent (PC) of a photoelectrochemical cell as a function of the delay between a 400 nm pump of fixed intensity and a 1200 nm push of varying intensities per pulse.

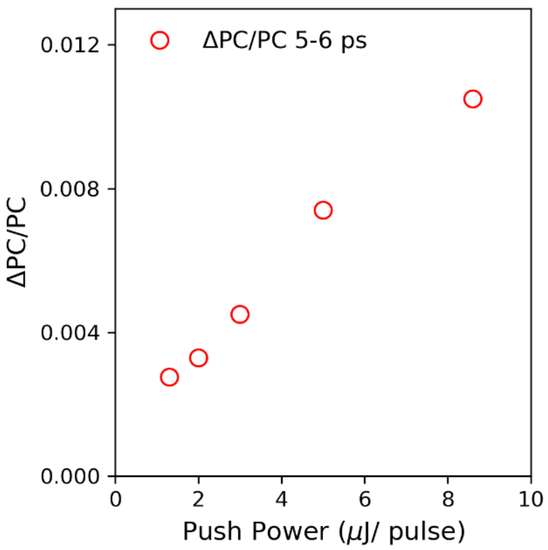

Supplementary Figure 5. Push intensity dependence of the PPPC signal. Pump-push-photocurrent (PPPC) response as a function of push power obtained by averaging the data from Supplementary Figure 4 between 5-6 ps. The photocurrent signal varies linearly with increasing resonant push intensity.

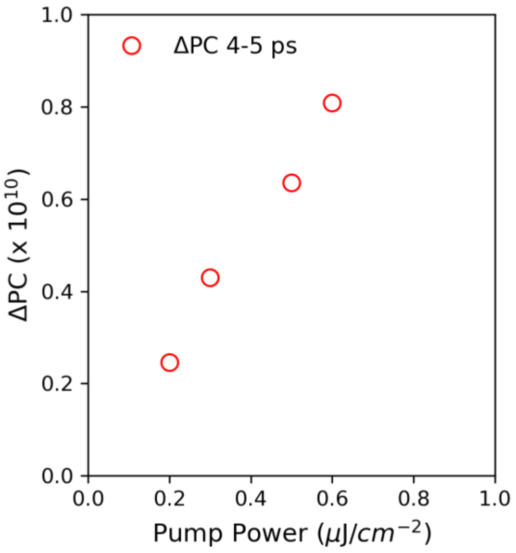

Supplementary Figure 6. Pump intensity dependence of the PPPC signal. Pump-push-photocurrent (PPPC) response as a function of pump power obtained by averaging the data from Figure 3a between 4-5 ps. The photocurrent signal varies linearly with increasing pump intensity. Note that the figure shows only the change in the push-induced current (ΔPC) as a function of pump-power.

a b

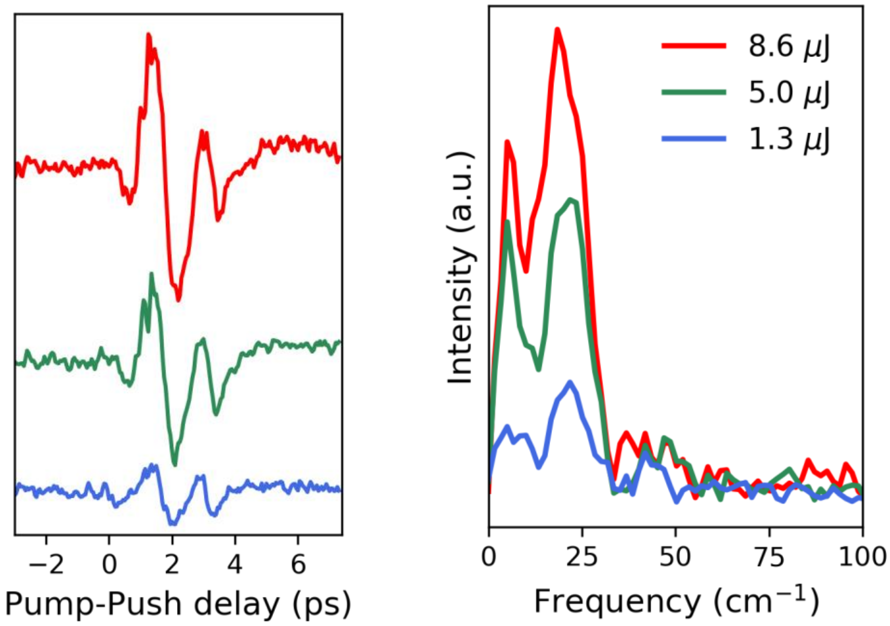

Supplementary Figure 7. Oscillation in the PPPC signal and Fourier Transform.
(**a**) Extracted oscillating component from the PPPC data shown in Supplementary Figure 4 and (**b**) Fourier Transform of such component.

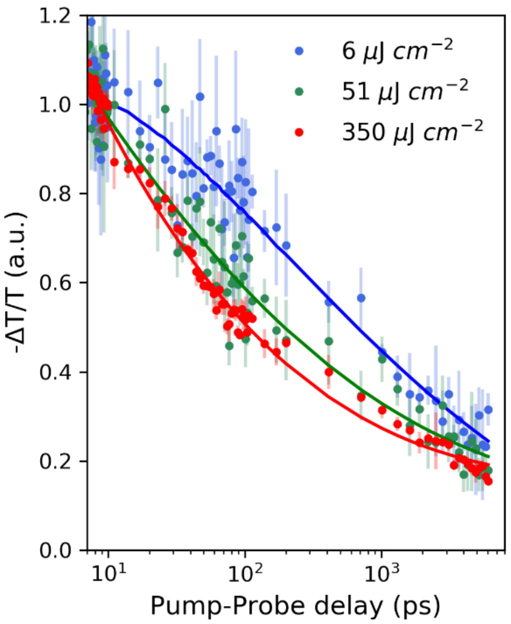

Supplementary Figure 8. **Fluence dependence of the TA at long times.** Normalised TA decay at long timescales at different pump powers showing a dependence of the decay kinetics on the density of charge carriers (coloured dots and standard deviation). This behavior is characteristic of bimolecular recombination (for example via a defect state) previously reported for α-Fe_2O_3 and other metal oxides.

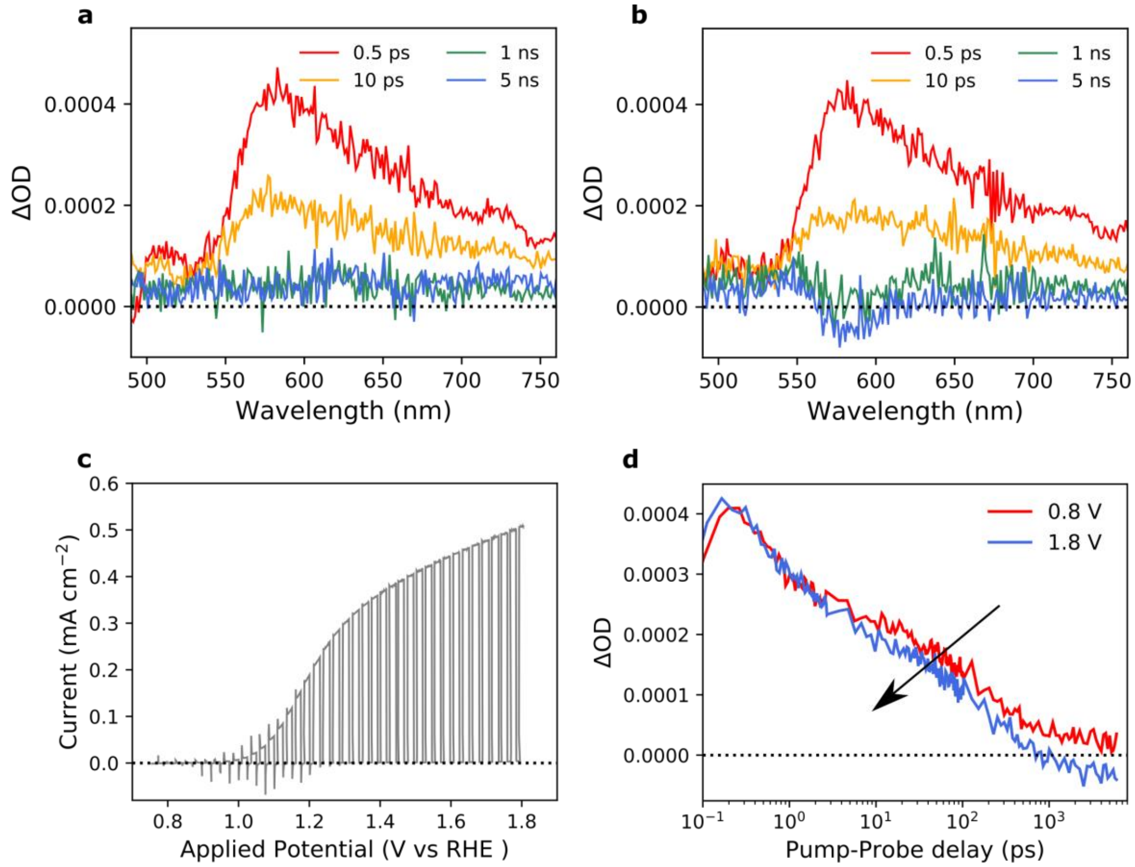

Supplementary Figure 9. Voltage dependence of the Transient Absorption. Transient Absorption spectra of the 20 nm ALD α-Fe_2O_3 photoanode in the visible region at an applied voltage of (**a**) 0.8 V vs RHE and (**b**) 1.8 V vs RHE. At strong applied potential we observe a change in sign of the main peak at ~575 nm. (**c**) Current-Voltage characteristic of the α-Fe_2O_3 photoanode in 1 M NaOH electrolyte solution under 1 sun illumination and (**d**) Kinetics of the TA peak in the visible (average between 570-578 nm). This data shows the same trend to the data we published in ([1]) for different Fe_2O_3 films. The observation of field dependent kinetics at longer timescales points towards a defect mediated recombination/trapping of the polarons formed at early timescales (< 2 ps). Similar to the intensity dependence data (Figure 3d and Supplementary Figure 8) we observe stronger field dependence after 100 ps.

Supplementary References

1. Pendlebury, S. R. *et al.* Ultrafast Charge Carrier Recombination and Trapping in Hematite Photoanodes under Applied Bias. *J. Am. Chem. Soc.* **136**, 9854–9857 (2014).
